# Recurrence Rate and Associated Factors of Mucinous Borderline Ovarian Tumors and Mucinous Ovarian Carcinomas: A Retrospective Study in the South of Vietnam

**DOI:** 10.3390/diagnostics16040562

**Published:** 2026-02-13

**Authors:** Tuan M. Vo, Ha Le, Uyen Huynh, Thuy L. Tran, Nhinh V. Chau, Nam H. Nguyen

**Affiliations:** 1 Faculty of Medicine, University of Medicine and Pharmacy at Ho Chi Minh City, Ho Chi Minh City 7000, Vietnam; vominhtuan@ump.edu.vn (T.M.V.); lthha.ths.san@ump.edu.vn (H.L.); tranlethuy@ump.edu.vn (T.L.T.); 2 OB-GYN department, University Medical Center, Ho Chi Minh City 7000, Vietnam; nhin.cv@umc.edu.vn

**Keywords:** mucinous borderline ovarian tumors, mucinous ovarian carcinomas, recurrence, risk factors

## Abstract

**Introduction:** Mucinous ovarian tumors are the second most common type of epithelial ovarian tumors. This study aimed to determine the recurrence rates and risk factors for mucinous borderline ovarian tumors (MBOTs) and mucinous ovarian carcinomas (MOCs). **Methods:** A retrospective study was conducted on 188 patients at Tu Du Hospital, Vietnam, from May 2019 to March 2023, with follow-ups until August 2024. The recurrence rates were calculated using life tables, while associated factors were analyzed through the log-rank test and Cox proportional hazards model. **Results:** The median time to recurrence was 13 months (range: 9–19 months), with twelve patients experiencing a recurrence. Overall cumulative recurrence rates (both MBOT and MOC) were 2.66% at 12 months, 5.32% at 24 months, and 6.45% at 36, 48, and 60 months. In the MBOT group, the recurrence rate was 5.80% at 60 months, while in the MOC group, it was 7.65% at 60 months. A significant relationship was found between higher recurrence rates and larger tumor size (1 cm increase resulted in a 10% risk reduction, HR = 0.90, *p* < 0.05), advanced FIGO stages (Stage III compared to Stage I, HR = 16.07, *p* < 0.05), and capsule rupture (HR = 6.79, *p* < 0.05). **Conclusions:** The total recurrence rate of MBOT and MOC after 60 months in Southern Vietnam was 6.45%. It is crucial to adopt follow-up strategies for high-risk patients to ensure early detection and treatment of recurrences. Additional studies with longer follow-up are necessary to identify late recurrences.

## 1. Introduction

Mucinous ovarian tumors, the second most common type among epithelial–stromal ovarian tumors after serous tumors, are characterized by mucin-secreting epithelial cells resembling either the endocervical-type (also known as Müllerian type) or gastrointestinal-type (intestinal or gastric type) epithelium. Approximately 80% of mucinous ovarian tumors are benign, 10% are borderline, and 10% are malignant carcinomas [1]. In the United States and Europe, mucinous ovarian tumors generally account for 12% to 15% of all ovarian tumors; however, the incidence appears to be higher in Japan [1]. Borderline mucinous ovarian tumors rank second in frequency in North America and Europe (accounting for 30–50% of all borderline tumors) but represent the most common subtype in Asia, comprising approximately 70% [2]. Mucinous ovarian carcinoma, or mucinous carcinoma, accounts for approximately 12% of malignant ovarian tumors. However, recent studies indicate that the actual incidence is around 3–4% [3].

The mean age of patients with MBOT and MOC typically ranges from 40 to under 60 years, whereas patients with benign mucinous tumors tend to be slightly younger [4,5,6]. In contrast to other epithelial ovarian tumors, borderline mucinous tumors may occur in adolescents or young adults, although the incidence in these age groups remains low. Mucinous tumors are characterized by large tumor size. These tumors often present as large masses, which can lead to urinary disturbances and compression of adjacent organs. Persistent recurrence after surgical treatment, with progressive histological changes from benign to borderline and malignant forms, remains a topic of ongoing debate. The phenomenon of persistent recurrence after surgical treatment, characterized by histological progression from benign to borderline and then to malignant lesions, remains a subject of considerable debate. In particular, the association with KRAS and TP53 gene mutations has attracted significant attention in recent international studies [3,7].

MOC is a distinct histological subtype of ovarian carcinoma, accounting for 5% of all [7]. While 80% of primary MOC cases are typically diagnosed at FIGO Stage I with a high overall survival (OS) rate [8,9], patients with recurrent disease have a very poor prognosis and show limited response to chemotherapy. Specifically, the 5-year survival rate for primary cases is 84.5%, while the median survival after recurrence is only 5.0 months [8,9]. Therefore, advanced-stage or recurrent MOC is a significant therapeutic challenge for clinicians.

Currently, prognostic factors for recurrence remain a topic of debate due to the limited number of studies. Key factors include age at diagnosis, FIGO stage, type of initial surgery (conservative or radical), tumor differentiation, immunohistochemical findings, tumor capsule integrity, and the presence of residual tumor after surgery [10,11,12,13,14]. Accurately determining the disease stage and identifying recurrence risk factors are essential. This helps guide treatment decisions, provides useful information for patient counseling, and enables better management to improve OS.

Tu Du Hospital is a leading institution in Southern Vietnam that focuses on obstetrics and gynecology, with a particular emphasis on gynecological cancer. There has been an increase in cases of mucinous ovarian tumors, with over 150 cases documented annually. Of these, approximately 40 to 50 cases are classified as MBOT and MOC. Current research on mucinous ovarian tumors in Southeast Asia, particularly in Vietnam, is rather scarce. The majority of available studies are part of broader research initiatives concerning ovarian tumors or ovarian carcinoma. This study aims to provide insights into the clinical and paraclinical features, treatment methods, recurrence rates, and associated factors regarding the recurrence of these tumors.

## 2. Methodology

### 2.1. Study Design and Participants

We conducted a retrospective study of patients diagnosed with mucinous borderline ovarian tumors (MBOT) and mucinous ovarian carcinoma (MOC) at the Gynecological Oncology Department of Tu Du Hospital in Ho Chi Minh City, Vietnam, between 3 May 2019 and 16 March 2023. Currently, all borderline ovarian tumors, including MBOT, fall under the ovarian cancer category according to ICD-10 code C56, which is consistent with other epithelial ovarian cancers. Our research encompassed all patients classified under ICD-10 code C56, whose initial surgical histopathological diagnosis was recorded as MBOT (ICD-O code 8472/1) or MOC (ICD-O code 8480/3). We excluded patients from the study based on two criteria: (i) the existence of mixed-type ovarian cancer or metastatic cancer and (ii) medical records that were incomplete and did not provide sufficient information for analysis.

### 2.2. Data Collection

We gathered demographic and clinicopathological information from the patients’ medical records. During the study period, a total of 221 patients with mucinous ovarian tumors were identified, of whom 33 were excluded, resulting in 188 patients included in the final analysis. The disease staging was established according to the 2014 guidelines set forth by the International Federation of Gynecology and Obstetrics (FIGO), utilizing surgical notes and pathological reports. The histological classification adhered to the 2020 World Health Organization (WHO) classification for tumors of the female genital tract.

MBOT and MOC recurrence was defined as the reappearance of disease in patients who had a histopathological diagnosis, underwent surgical treatment, achieved complete response at discharge, and subsequently met at least one of the following criteria: (i) a rise in CA 125 levels consistent with Rustin’s criteria [7]; (ii) clinical or imaging evidence of disease recurrence; or (iii) histopathological confirmation of recurrence in surgically treated cases. According to Rustin’s criteria, CA 125-based recurrence was defined as (i) CA 125 levels exceeding twice the upper limit of normal on two separate occasions at least one week apart, in patients whose CA 125 levels normalized after treatment, or (ii) CA 125 levels exceeding twice the nadir value on two occasions at least one week apart, in patients whose CA 125 levels remained elevated post-treatment without normalization. The normal cut-off value for serum CA-125 was <35 U/mL. Given the known limited sensitivity and inconsistent elevation of CA-125 in mucinous ovarian tumors, CA-125 was used only as an adjunctive follow-up parameter and was always interpreted in combination with clinical findings, imaging studies, or histopathological confirmation. At Tu Du Hospital, patients treated for MOC and MBOT were followed monthly for the first 6 months, every 2 months for the next 12 months, every 3 months for the subsequent 18 months, every 6 months for the following 24 months (completing a 5-year period), and annually thereafter. Follow-up data were updated through August 2024 based on a review of medical records. Time to recurrence (in months) was defined as the interval from completion of MOC and MBOT treatment to the date of confirmed recurrence. For patients without recurrence, follow-up time was calculated from treatment completion to the date of last follow-up, end of study, or death. Using the sample size formula for survival analysis with a hazard ratio (HR) of 3.84—based on the previous study by Hiroaki Kajiyama et al. (2019) [14]—a significance level (α) of 0.05, and statistical power (1 − β) of 0.90, the minimum required number of recurrence events was estimated to be 12. Recurrence rates in MBOT and MOC vary by disease stage, typically ranging from 18% to 45%. To obtain the largest sample size requirement, a prevalence (Prev) of 0.18 was selected. Accordingly, the minimum total number of MBOT and MOC cases required for analysis was calculated to be 64.

### 2.3. Data Analysis

Data analysis was performed with Stata version 17.0. Descriptive statistics, including percentages and medians, were employed to evaluate demographic and clinicopathological characteristics. To investigate the relationship between categorical variables and recurrence time, univariate analyses were conducted using the log-rank test for equality. Following this, we utilized Cox proportional hazards regression for multivariate analyses to control for possible confounding factors. The variables selected for the multivariate models were based on their bivariate associations (*p* < 0.25). A *p*-value of less than 0.05 was considered statistically significant.

## 3. Results

Between May 2019 and March 2023, a total of 221 cases of Müllerian borderline tumor (MBOT) and malignant ovarian carcinoma (MOC) were recorded. Among these cases, 27 were noted to have inadequate medical documentation, 4 were identified as mixed-type carcinoma featuring components of endometrioid adenocarcinoma, 1 case pertained to an MBOT located in the fallopian tube, and 1 case involved a secondary MOC that had metastasized from the gastrointestinal tract.

The mean age of patients at their first surgery was 41.4 ± 15.8 years, with a range of 16 to 85 years. Most patients were of Kinh ethnicity (97.9%), and 76.6% were premenopausal. Preoperative ROMA test results revealed that 38.8% were at high risk, while the remaining were at low risk. Bilateral tumors were found in 3.7% of cases, with the rest having unilateral tumors (96.3%). Of the surgeries, 86.7% were laparotomies. Tumor rupture occurred in 38.8% of cases before or during surgery, 42.0% had abdominal fluid during surgery, and 10.1% had residual tumor postoperatively. The average tumor size in pathological samples was 195.6 ± 99.0 mm, with the largest measuring 550 mm. FIGO Stage I was observed in 92.5% of cases, Stage II in 4.3%, and Stage III in 3.2%, with no cases of Stage IV. Histological analysis showed 64.9% cases with MBOT and 35.1% cases with MOC, with 34.6% showing stromal invasion.

The study recorded 73 cases undergoing intraoperative frozen section (IFS) (38.8%). Among these, 16 cases were diagnosed as benign (21.9%), 40 cases as borderline (54.8%), and 17 cases as carcinoma (23.3%). Accordingly, the sensitivity of IFS in diagnosing MBOT and MOC was 78.1% [95% CI: 0.67–0.87]. The false-negative rate for IFS was 21.9% [95% CI: 0.12–0.31] (Table 1).

The median follow-up time was 46 months (interquartile range: 27–54 months). In the recurrence group, the median time to recurrence was 13 months, with an interquartile range of 9–19 months. The estimated cumulative risk of recurrence for both MBOT and MOC at 12, 24, and 36 months was 2.66%, 5.32%, and 6.45%, respectively (Table 2).

When analyzed separately by histopathological subtype, the recurrence rate was 5.80% in the MBOT group and 7.65% in the MOC group (Table 3 and Table 4). Specifically, over time, the estimated cumulative risk of recurrence for MBOT at 12, 24, and 36 months was 3.28%, 4.92%, and 5.80%, respectively. In contrast, for MOC, the corresponding values were 1.52%, 6.06%, and 7.65%, respectively.

We performed univariate Cox regression analysis for 15 independent variables (age at diagnosis, BMI, menstrual status, history of benign ovarian tumor, ROMA test, tumor laterality, tumor integrity, ascites, surgical method, type of surgery, tumor size, residual disease after surgery, FIGO stage, histological subtype (MBOT vs. MOC), and stromal invasion). After the univariate analysis, multivariate Cox regression was conducted for six factors with *p* < 0.25 (history of benign ovarian tumor, ROMA test, tumor size, tumor integrity, residual disease after surgery, and FIGO stage) to control for potential confounding and interaction effects. In addition, histopathological subtype, an important prognostic variable, was also included in the multivariate model. In the multivariate Cox regression model (Table 5), three factors related to recurrence were found to be statistically significant (*p* < 0.05): tumor size, tumor integrity, and FIGO stage.

For every 1 cm increase in tumor size, the risk of recurrence decreased by 10%, with a hazard ratio (HR) of 0.90 [95% CI: 0.81–0.99]. Tumor rupture before and during surgery was associated with a 6.79-fold increased risk of recurrence [95% CI: 1.29–35.54] compared to patients without tumor rupture. Patients with MBOT and MOC at Stage III or higher had a 16.07-fold increased risk of recurrence [95% CI: 2.80–92.18] compared to those at Stage I.

The plots illustrated below were constructed based on the disease-free survival function over time. The disease-free survival function at a given time point *t* represents the probability that a subject remains free of the event (recurrence) at time *t*. We only present Kaplan–Meier curves of recurrence-free survival for factors that showed statistical significance in the multivariate Cox regression model, specifically tumor integrity and FIGO stage.

**Figure 1:**
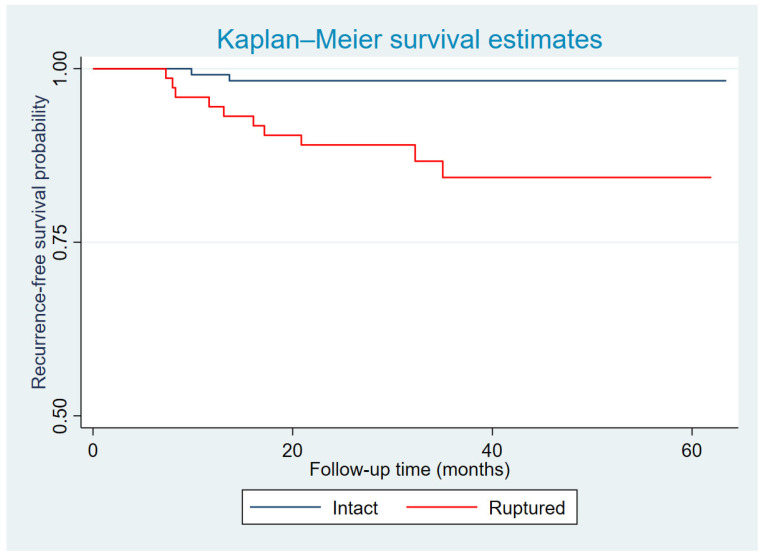
Kaplan–Meier curve of recurrence-free survival probability according to tumor integrity.

At 1 year, the recurrence-free survival rates were 99.1% in the intact capsule group and 94.5% in the ruptured capsule group. At 3 years, these rates were 98.3% and 84.3%, respectively, and the rates remained stable up to 5 years of follow-up. Patients with ruptured capsules had a markedly lower recurrence-free survival rate compared with those with intact capsules (Figure 1).

**Figure 2:**
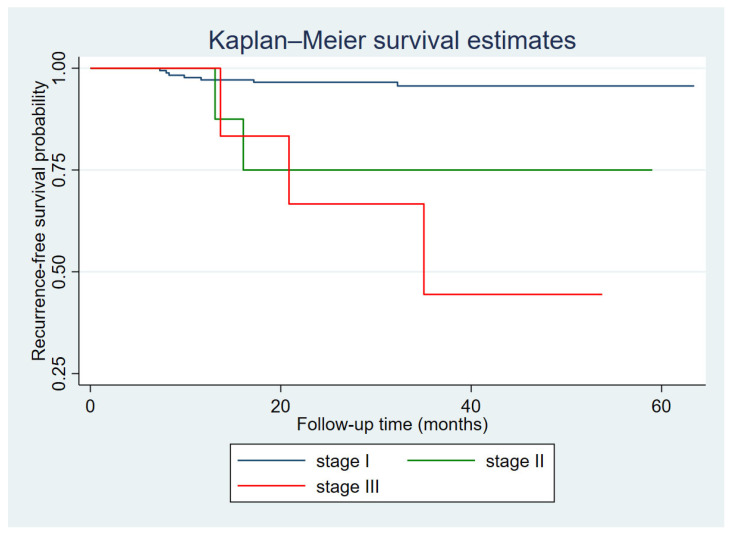
Kaplan–Meier curve of recurrence-free survival probability according to FIGO stage.

At 1 year, the recurrence-free survival rates were 97.1% in patients with Stage I disease and 100% in those with Stage III disease. At 3 years, these rates were 95.6% and 44.4%, respectively, and remained unchanged thereafter. Patients with Stage III disease had a significantly lower recurrence-free survival compared with those with Stage I disease (Figure 2).

## 4. Discussion

Our research monitored 188 patients diagnosed with MBOT and MOC over a median period of 46 months, during which there were 12 cases of recurrence, occurring at a median interval of 13 months (interquartile range: 9–19 months). The cumulative recurrence risk at the 60-month mark was 6.45%. Recurrence rates were recorded at 2.66% at 12 months, 5.32% at 24 months, and 6.45% at 36 months. This rate remained consistent up to 60 months. This suggests that 83.3% of recurrences occurred within the first two years, while only 16.6% occurred in the third year. No new recurrences were noted between the fourth and fifth years of follow-up.

This recurrence rate is lower than that reported in several previous studies, including 11.8% in the study by Li Sun (2018) [13], 9.3% in the report by Kumari S (2021) [15], and 9% over the first five years of observation in the study by Robert L.H. (2021) [8]. The differences in recurrence rate may be attributed to variations in patient populations and predominant surgical approaches. Our study included both borderline ovarian tumors and ovarian carcinomas, but most cases were diagnosed at an early stage. The study by Kenneth et al. in the United States (2000) also reported an overall recurrence risk of 8% for both MOC and MBOT groups, which is quite consistent with our findings. The author additionally calculated the recurrence risk for each histopathological subgroup after determining the overall rate [16]. Notably, the rate of cystectomy alone in our cohort was 11.7%, which is lower than that reported in other studies.

The sensitivity of intraoperative frozen section (IFS) in our study for MBOT and MOC was 78.1%, which is comparable to the results reported by Jeong Y.P. (2019) (78.9%) [17] and Wen Z. (2019) [18]. However, the IFS of MBOT and MOC has been shown to be less accurate compared to other epithelial ovarian tumors, as previously reported [19,20,21]. The sensitivity of IFS for borderline tumors generally ranges from 67 to 74%, while for MOC, it is approximately 55–72%. In contrast, other epithelial ovarian tumors, especially serous tumors, have considerably higher accuracy rates, reaching 90–97%. This discrepancy may be explained by the large size of mucinous ovarian tumors, their inherent heterogeneity, and the coexistence of benign, borderline, and malignant components within the same mass, which increases the likelihood of sampling error and misrepresentation of the true nature of the tumor. Therefore, pathologists have recommended that, in mucinous ovarian tumors, multiple samples should be obtained from different areas, particularly from suspicious sites such as the solid component, papillary projections, mural nodules, or thickened septa, to minimize sampling error and improve the diagnostic accuracy of IFS [19,20].

Furthermore, over 80% of patients in our study underwent a second surgery for adnexectomy and staging following initial cystectomy. In contrast, other studies primarily focused on borderline ovarian tumors, with higher rates of fertility-sparing surgery. This surgical approach has been associated with an increased recurrence risk, as reported in the literature. The median follow-up time in our study (46 months) was shorter than that reported in some other studies (151 months in the study by Li Sun [13]), which may explain why late recurrences beyond 5 years have not yet been detected.

In the study by Tuan VM (2017) [21], which included 433 patients who underwent surgery for borderline malignant ovarian tumors at Tu Du Hospital, the median time to recurrence was 18 months. The median recurrence time reported by Tuan VM was longer than observed in our study (18 months vs. 13 months), likely because his study included all borderline malignant ovarian tumors, whereas our study focused on malignant mucinous ovarian tumors (including both borderline and carcinoma cases), which may have led to a shorter recurrence interval.

Our results demonstrated that for each 1 cm increase in tumor size, the risk of recurrence decreased by 10%, with HR = 0.90 [95% CI: 0.81–0.99]. The impact of tumor size on recurrence remains inconclusive; however, several studies have suggested that a smaller tumor size is associated with a higher risk of recurrence. In a study from Thailand, Phansenee S (2021) [22] reported that, among 168 patients with borderline ovarian tumors, tumors < 10 cm were associated with poorer survival outcomes and a higher risk of disease progression compared with tumors ≥ 10 cm (*p* = 0.03). One of the most important factors influencing recurrence risk is the extent of radical surgery and complete staging. Evidence shows that incomplete surgery is the strongest predictor of recurrence: if residual tumor remains or overly conservative surgery is performed, recurrence rates can reach 10–20%, compared with about 5% after initial radical surgery [23,24,25]. Smaller tumors are sometimes underestimated in terms of malignancy, which may lead to more conservative approaches. For borderline mucinous ovarian tumors, cystectomy for fertility preservation has been shown to significantly increase the recurrence risk. This is because mucinous tumors are often multifocal, with benign, borderline, and carcinoma areas coexisting within the same mass. Cystectomy may leave microscopic disease behind or allow regrowth from residual ovarian tissue. Our study found that patients with malignant mucinous ovarian tumors at Stage III or higher had a 16.07-fold increased risk of recurrence compared with those at Stage I, and this difference was statistically significant. Disease stage is an important prognostic factor for tumor recurrence, as has been consistently confirmed in the literature through studies conducted both internationally and in Vietnam [8,11,21,26,27]. The substantial difference in recurrence risk between Stage III and Stage I malignant mucinous ovarian tumors can be explained by several factors related to pathological characteristics and treatment. At Stage III, the tumor has already extended beyond the ovary, frequently disseminating to the peritoneum or involving lymph node metastasis. This makes complete cytoreductive surgery more challenging. Even with maximal effort toward optimal debulking, the likelihood of microscopic residual disease in Stage III patients remains higher compared with those at Stage I. Furthermore, from a tumor pathobiology and adjuvant chemotherapy perspective, mucinous ovarian carcinoma exhibits relatively lower sensitivity to platinum-based chemotherapy, the standard regimen for ovarian cancer, compared with other histological subtypes.

Our research indicated that intraoperative tumor rupture was linked to a 6.79-fold higher risk of recurrence compared to cases without rupture, with this variance being statistically significant. The commonly accepted explanation is that when the tumor capsule is ruptured, cancerous cells can escape into the peritoneal cavity, where they may attach and create small deposits that cannot be eliminated through surgery. These small foci can later evolve into recurrent tumors in the abdominal or pelvic areas. As a result, preserving the tumor’s integrity during surgical procedures is crucial to reducing the likelihood of recurrence.

### Limitations of the Study

This study was conducted retrospectively, utilizing data gathered from medical records that were not entirely standardized, which may introduce information bias. For patients who were lost to follow-up or did not attend scheduled appointments, the survival analysis model accommodates a certain percentage of censored cases. Additionally, patient compliance with follow-up at the Department of Gynecologic Oncology was generally high due to the characteristics of cancer treatment. Furthermore, the median follow-up period in this research was 46 months. Mucinous ovarian carcinoma (MOC) and borderline ovarian tumors (MBOTs) typically present at an early stage and have a low early recurrence rate; however, the risk of late recurrence still exists, potentially occurring over 10 years after the initial treatment.

## 5. Conclusions

Patients diagnosed with MOC and MBOT who display increased recurrence risk factors necessitate comprehensive counseling and careful monitoring, particularly in cases involving intraoperative tumor rupture and those classified as Stage III or more advanced. It should be noted that smaller tumor sizes are associated with an increased likelihood of recurrence. Furthermore, our study found that the false-negative rate for frozen sections in MOC and MBOT was 21.9%. As a result, intraoperative decisions should not solely rely on frozen section results; routine histopathological evaluation of the tumor is essential.

## Figures and Tables

**Table 1:** Clinical and histopathologic characteristics of patients with MOC and MBOT.

Characteristics	Total	Non-Recurrences (n = 176)	Recurrences (n = 12)	*p*-Value *
	n (%)	n (%)	n (%)	
**Age at diagnosis (years)**				
>50	57 (30.3)	52 (27.6)	5 (2.7)	0.56
35–50	62 (33.0)	59 (31.4)	3 (1.6)	0.74
<35	69 (36.7)	65 (34.6)	4 (2.1)	
**BMI**				
Underweight	16 (8.5)	15 (8.0)	1 (0.5)	0.85
Overweight/Obese	71 (37.8)	65 (34.6)	6 (3.2)	0.38
Normal	101 (53.7)	96 (51.1)	5 (2.6)	
**Menstrual status**				
Menopausal	44 (23.4)	41 (21.8)	3 (1.6)	0.93
Premenopausal	144 (76.6)	135 (71.8)	9 (4.8)	
**History of benign ovarian tumor**				
Yes	19 (10.1)	16 (8.5)	3 (1.6)	0.09
No	169 (89.9)	160 (85.1)	9 (4.8)	
**ROMA test**				
High risk	73 (38.8)	66 (35.1)	7 (3.7)	0.17
Low risk	115 (61.2)	110 (58.5)	5 (2.7)	
**Tumor laterality**				
Bilateral	7 (3.7)	6 (3.2)	1 (0.5)	0.42
Unilateral	181 (96.3)	170 (90.4)	11 (5.9)	
**Tumor integrity**				
Ruptured	73 (38.8)	63 (33.5)	10 (5.3)	**0.01**
Intact	115 (61.2)	113 (60.1)	2 (1.1)	
**Ascites**				
Present	109 (58.0)	104 (55.3)	5 (2.6)	0.28
Absent	79 (42.0)	72 (38.3)	7 (3.7)	
**Surgical method**				
Laparoscopic	25 (13.3)	23 (12.2)	2 (10.6)	0.79
Open surgery	163 (86.7)	153 (81.4)	10 (5.3)	
**Type of surgery**				
Conservative	95 (50.5)	90 (47.9)	5 (2.7)	0.57
Radical	93 (49.5)	86 (45.7)	7 (3.7)	
Tumor size (cm) **	**19.6 ± 9.9**	19.9 ± 9.9	14.4 ± 8.4	0.07
**Residual disease after surgery**				
Yes	19 (10.1)	15 (8.0)	4 (2.1)	**0.01**
No	169 (89.9)	161 (85.6)	8 (4.3)	
**FIGO stage**				
Stage III	6 (3.2)	3 (1.6)	3 (1.6)	**0.01**
Stage II	8 (4.3)	6 (3.2)	2 (1.1)	**0.02**
Stage I	174 (92.5)	167 (88.8)	7 (3.7)	
**Intraoperative frozen-section analysis (IFS)**				
Yes	73 (38.8)	72 (38.3)	1 (0.5)	
No	115 (61.2)	104 (55.3)	11 (5.9)	
**IFS results (n = 73)**				
Benign	16 (21.9)	15 (20.5)	1 (1.4)	
MBOT or MOC	57 (78.1)	57 (78.1)	0 (0)	
**Histological type**				
MOC	66 (35.1)	61 (32.4)	5 (2.7)	0.64
MBOT	122 (64.9)	115 (61.2)	7 (3.7)	
**Stromal invasion**				
Present	65 (34.6)	59 (31.4)	6 (3.2)	0.26
Absent	123 (65.4)	117 (62.2)	6 (3.2)	

* p-value from log-rank test results; ** continuous variable is presented as mean ± standard deviation.

**Table 2:** Risk of overall recurrence over time.

Time Interval (Months)	Non-Recurrences Entering the Interval	Recurrences (n = 12)	Censored Cases	Risk of Recurrence (%)	Cumulative Risk of Recurrence (95%CI)
0–12	188	5	0	2.66	2.66 (1.12–6.27)
12–24	183	5	11	2.73	5.32 (2.90–9.66)
24–36	167	2	54	1.20	6.45 (3.72–11.09)
36–48	111	0	37	0.00	6.45 (3.72–11.09)
48–60	74	0	63	0.00	6.45 (3.72–11.09)
>60	11	0	11	0.00	6.45 (3.72–11.09)

**Table 3:** Risk of recurrence over time in the borderline mucinous ovarian tumor group.

Time Interval (Months)	Non-Recurrences Entering the Interval	Recurrences (n = 12)	Censored Cases	Risk of Recurrence (%)	Cumulative Risk of Recurrence (95%CI)
0–12	122	4	0	3.28	3.28 (1.24–8.50)
12–24	118	2	8	1.06	4.92 (2.24–10.62)
24–36	108	1	35	0.93	5.80 (2.81–11.78)
36–48	72	0	24	0.00	5.80 (2.81–11.78)
48–60	48	0	47	0.00	5.80 (2.81–11.78)
>60	1	0	1	0.00	5.80 (2.81–11.78)

**Table 4:** Risk of recurrence over time in the mucinous ovarian carcinoma group.

Time Interval (Months)	Non-Recurrences Entering the Interval	Recurrences (n = 12)	Censored Cases	Risk of Recurrence (%)	Cumulative Risk of Recurrence (95%CI)
0–12	66	1	0	1.52	1.52 (0.21–10.27)
12–24	65	3	3	4.62	6.06 (2.32–15.35)
24–36	59	1	19	1.69	7.65 (3.26–11.42)
36–48	39	0	13	0.00	7.65 (3.26–11.42)
48–60	26	0	16	0.00	7.65 (3.26–11.42)
>60	10	0	10	0.00	7.65 (3.26–11.42)

**Table 5:** Multivariate regression analysis of factors associated with recurrence.

Factors	Risk Time (Months)	Recurrences		Cox Proportional Hazards Regression, HR (95% CI)			
		n/Total	Incidence Rate	Univariate Analysis	*p* *	Multivariate Analysis	*p* **
**History of benign ovarian tumor**							
Yes	742	3/19	4.04	3.09	0.09	1.83	0.44
No	7054	9/169	1.27	1		1	
**Histological type**							
MOC	2790	5/66	1.79	1,32	0.64	1.13	0,85
MBOT	5006	7/176	1.40	1		1	
**ROMA test**							
High risk	3054	7/73	2.29	2.23	0.17	0.94	0.94
Low risk	4742	5/115	1.05	1		1	
**Tumor size (cm)**	7796	12/188	1.54	0.93	0.07	0.90	**0.04**
**Tumor integrity**							
Rupture	2802	10/73	3.57	8.53	0.01	6.79	**0.02**
Intact	4994	2/115	0.40	1		1	
**Residual disease after surgery**							
Yes	723	4/19	5.53	4.66	0.01	1.14	0.85
No	7073	8/169	1.13	1		1	
**FIGO stage**							
Stage III	311	3/6	9.06	13.16	**<0.01**	16.07	**<0.01**
Stage II	315	2/8	6.35	6.49	0.02	3.06	0.25
Stag I	7170	7/174	0.98	1		1	

* p-value of univariate analysis; ** p-value of multivariate analysis. CI: confidence interval; HR: hazard ratio.

## Data Availability

The datasets generated during and/or analyzed during the current study are available from the corresponding author upon reasonable reques.
